# Prevalence of carpal tunnel syndrome among dentists: a systematic review and meta-analysis

**DOI:** 10.12688/f1000research.131173.2

**Published:** 2023-03-20

**Authors:** Evangelos Kostares, Georgia Kostare, Michael Kostares, Maria Kantzanou

**Affiliations:** 1 School of Health Sciences, National and Kapodistrian University, Athens, 11527, Greece

**Keywords:** carpal tunnel syndrome, CTS, entrapment neuropathy, dentists, dental surgeons, prevalence, meta-analysis

## Abstract

**Purpose:** To estimate the prevalence of carpal tunnel syndrome (CTS) among dental surgeons and the effect of possible moderators on it.

**Methods:** A systematic literature search (Medline and Embase databases) was conducted independently by two reviewers. Only observational studies that examined specifically the prevalence rates of CTS among dentists were included. Quality assessment was performed. The pooled prevalence with 95% confidence intervals (CI) was estimated. Outlier and influential analysis were conducted. Moderator analysis was performed in order the effect of categorical and continuous variables on the estimated prevalence to be investigated.

**Results:** In total, ten eligible studies (3,547 participants) were finally included in this meta-analysis. Two of them were estimated as high quality (low risk of bias) and the remaining ones as moderate quality (moderate risk of bias). The overall prevalence of CTS among dental surgeons was estimated as 9.87% (95%CI 6.84%-14.03%) with significant heterogeneity between studies. No study was identified as influential. Potential sources of heterogeneity were not identified through the moderator analysis. In the subgroup analysis the prevalence was 12.47% (95%CI 6.38%-22.95%) for the group identified as having CTS through medical history and at least clinical examination or electrodiagnostic testing and 8.56% (95%CI 5.53%-13.01%) among those who identified solely through questionnaire (previously diagnosed).

**Conclusions:** Our findings are important to provide the pooled prevalence of CTS among dentists. Our results were based on highly heterogeneous studies. Our study reports a considerable prevalence, consequently, significance of awareness among dental surgeons regarding the etiology of this issue is more than necessary. More studies need to be conducted that could guide researchers in order this issue to be fully investigated.

## Introduction

Carpal tunnel syndrome (CTS) is one of the most frequent and well-studied entrapment neuropathies with a prevalence in middle-aged general population estimated at around 4.0% to 5.0%. ^12^ As per its’ pathophysiology, CTS occurs as the median nerve is being compressed and damaged through its passage within the narrow osteofibrous canal (carpal tunnel). ^27^^,^^34^ Among the great variety of symptoms that may occur, CTS is also identified by patients as pain, paraesthesias (especially, during the night) and dysaesthesias in the distribution of the median nerve (in the first three and a half digits of the affected hand), are the predominant ones. As CTS pathophysiology evolving, all muscles innervated by branches of the median nerve (flexor pollicis brevis, abductor pollicis brevis, opponens pollicis) are being atrophied and weakened, resulting the patient’s declined functionality. ^9^^,^^14^^,^^23^^,^^24^ On a regular basis, the diagnosis of CTS can be made throughout the combination of a comprehensive patient’s history and a thorough clinical examination (including Tinel, Phalen and Durkan’s tests). Yet, in specific patients, advanced procedures (electrodiagnostic tests) such as the nerve conduction studies, can be utilized both in the diagnosis as well as in treatment decision making. ^27^ Many risk factors have been identified throughout the years including the obesity, diabetes, hypothyroidism, pregnancy, lupus erythematosus and Reynaud’s phenomenon.

In the recent years, specific interest exists regarding the occurrence of CTS in certain occupations such as the dental surgeons, ^2^^,^^13^^,^^20^^,^^21^ that is expected to be higher than the general occupation ^31^ given that the procedures that are usually performed require the use of vibratory tools, strong griping, uncomfortable hand position and the performance of long-lasting repetitive tasks. ^29^

Therefore, the aim of this study is to review the available literature for data related to the occurrence of CTS in dental surgeons and to obtain an accurate estimate of its’ prevalence. On a secondary basis, an attempt to identify factors that may be associated with its’ prevalence will be performed.

## Methods

This review is reported in line with the PRISMA guidelines. ^38^

### Search strategy

A literature search of Medline (PubMed search engine) and Embase (Scopus search engine) database was conducted through inception up to December 16 ^th^, 2022, following the Preferred Reporting Items for Systematic Reviews and Meta-Analysis (PRISMA) guidelines. ^28^ The literature search was independently performed by two reviewers, using the following algorithm: (carpal tunnel syndrome OR CTS OR entrapment neuropathy OR median nerve compression) AND (“dentists” OR “dental surgeon”).

The reference lists of all identified eligible studies were evaluated by both reviewers for potentially missed articles from the initial literature search. Following the aforementioned procedure, all studies were stored in the Zotero reference management software (version 6.0.18) and the duplicate citations were removed. ^36^ The remaining articles were independently screened by two investigators to identify studies that met the pre-determined inclusion criteria. The study selection was conducted in two stages. First, article titles and abstracts were reviewed and those that did not meet our inclusion/exclusion criteria were removed. Secondly, the full texts of the remaining articles were retrieved and evaluated. If an absence in studies selection procedure was notified, the final decision was reached by team consensus.

### Criteria for study selection and data extraction

Articles that examined specifically the prevalence rates of CTS among dentists were included. Only observational studies written in English language were inserted with no restriction on publication date. Case reports, case series with less than ten participants, review articles, clinical trials, animals studies, letters to the editor, books, expert opinion, conference abstracts, studies with no full-text available, studies not written in English language, articles reported solely the prevalence of CTS’ symptoms, studies regarding dental laboratory technicians and dental hygienists were excluded. In articles with overlapping populations, the most recent or most complete publication was considered eligible. The following variables were obtained from each study: the first author’s name, year of publication, study design, continent of origin, study period, total number of patients, proportion of males, mean age, participants with CTS and diagnostic procedures.

### Quality assessment

Quality appraisal was independently performed by two investigators using the National Heart, Lung, and Blood Institute (NHLBI) Quality Assessment Tools. The NHLBI quality assessment tool for Observational Cohort and Cross-Sectional Studies was employed. Individual studies were assessed for potential flaws in accordance the study methodology or the conduct of each survey that could jeopardize internal validity. For each of the fourteen questions, investigators could select one of the following answers: “yes”, “no”, “cannot determine” (e.g. data were unclear or contradictory) or “not reported” (e.g. missed data) or “not applicable” (e.g. not relevant question regarding this type of study). Study quality was defined as “low”, “moderate” or “high” risk of bias. ^25^

### Statistical analysis

Statistical analysis was carried out using RStudio (version: 022.12.0+353) software (RStudio Team (2022)). ^32^ The meta-analysis was conducted through metafor package. ^33^ The DerSimonian and Laird random-effects model was used to estimate the pooled prevalence and its respective 95% confidence intervals (CI). Logit transformation was performed. Heterogeneity presence between studies was evaluated through visual inspection of the forest plot and by using the Cochran’s Q statistic and its respective p value. The Higgins I ^2^ statistic and its respective 95% CI were used for quantifying the magnitude of true heterogeneity in effect sizes. An I ^2^ value of 25%, 50%, and 75% indicated low, moderate, and high heterogeneity, respectively. To determine if the potential outlying effect sizes (as evaluated in the forest plot) were also influential, screening for externally studentized residuals with z-values larger than two in absolute value and leave-one-out diagnostics were performed. ^34^ Due to high heterogeneity remaining, a moderator analysis was performed. In the conducted subgroup analysis, the continent of origin and the diagnostic procedure (verified during the implementation of each study or previously diagnosed) were chosen as the categorical moderators on effect sizes. In the performed meta-regression analysis with continuous variables, the year of publication and the proportion of males were assessed as moderators on effect sizes. Owing to the limited availability of data (less than ten studies for each covariate) regarding other variables (e.g mean age, obesity, diabetes, hypothyroidism, pregnancy, autoimmune diseases), these data were not included in this analysis. ^17^ Unless otherwise stipulated, the statistical significance was established at p=0.05 (two-tailed). Tests to evaluate publication bias, such as Egger’s test, ^10^ Begg’s test ^5^ and funnel plots, were developed in the context of comparative data. They assume studies with positive results are more frequently published than studies with negative results, however in a meta-analysis of proportions there is no clear definition or consensus about what a positive result is. ^4^ Therefore, publication bias in this current meta-analysis was assessed qualitatively.

## Results

### Search results and characteristics of the included studies

**Figure 1.**
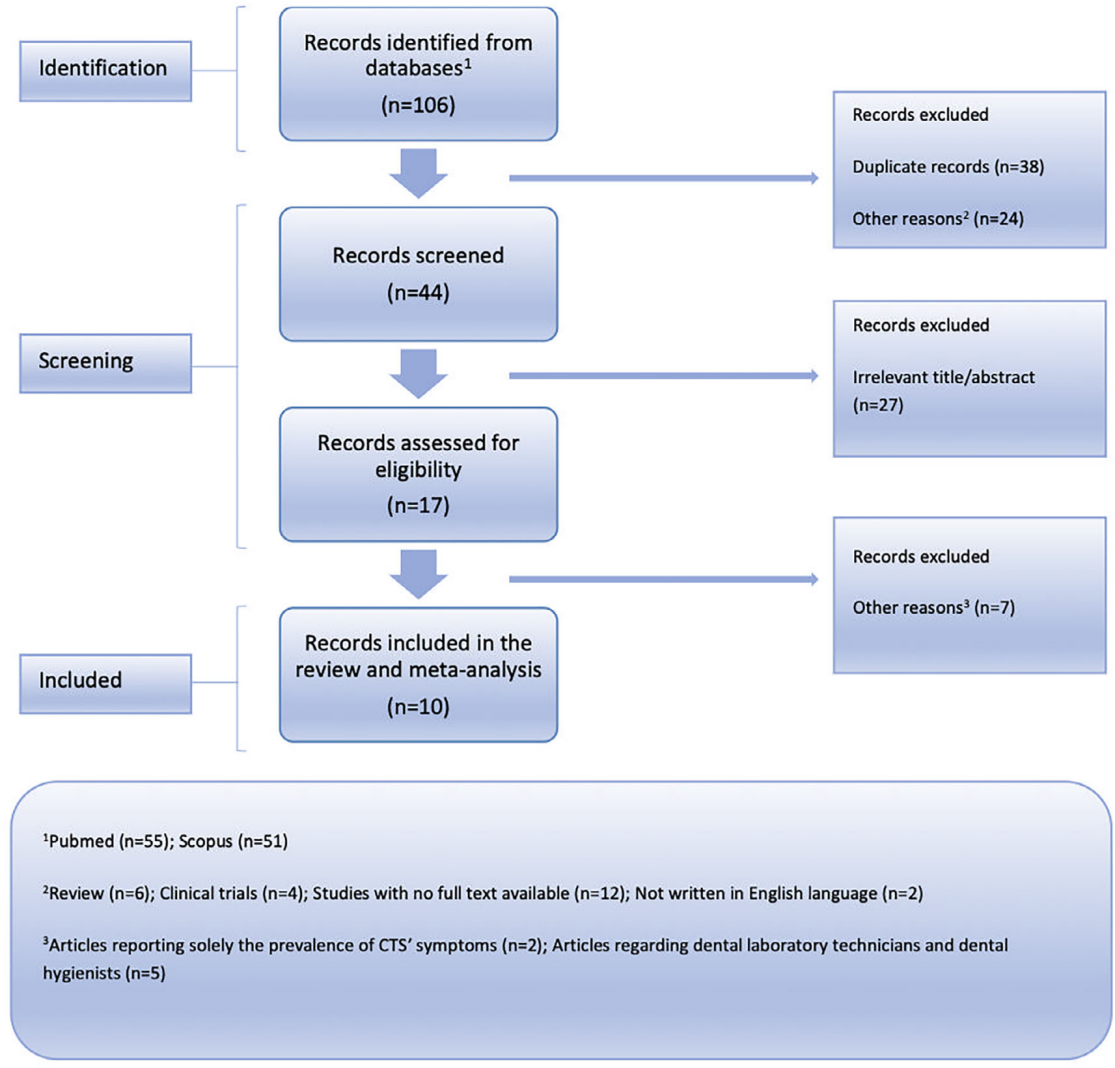
Flow chart depicting the systematic search results from the relevant studies' identification and selection.

As reported in the relevant section (Criteria for study selection and data extraction), manuscript that were only related to the prevalence of CTS’ symptoms (such as the study conducted from Prasad, D.A. *et al.* ^30^) and studies regarding dental hygienists (such as the study conducted from Anton D., *et al* ^3^ and Cherniack M., *et al.* ^8^) were excluded. In total, ten (n=10) eligible studies (3,547 participants) were finally included in this analysis (see Figure 1 for the PRISMA flow chart). ^37^ In 6 of the eligible studies CTS was diagnosed through questionnaire (patients previously diagnosed) and in the rest of them through medical history and at least clinical examination or electrodiagnostic testing. The descriptive characteristics of the incorporated research are presented in Table 1. All articles were published from 2001 to 2021 (conducted from 1997 to 2019). All of them were found to be of cross-sectional design. Most studies were contemplated in Asia (Iran, Lebanon, Saudi Arabia, n=6), followed by America (USA, Brazil, n=2) and Europe (Czech, Germany, n=2). The average percent of males was 54.22% while the mean age of participants ranged from 35 years to 46.4 years (median=38.2 years). Lastly, two studies were estimated as high quality (low risk of bias) and the remaining ones as moderate quality (moderate risk of bias).

**Table 1.** Main characteristics and data outcome of the included studies.

Author	Year of publication	Study design	Continent of origin	Study period	Total participants	Proportion of males	Mean age	CTS	Diagnosis	Quality assessment
Hamann C ^14^	2001	Cross-sectional	America (USA)	1997-1998	1079	83.6	NR	52	Q, EDT	Moderate
Haghighat A ^15^	2012	Cross-sectional	Asia (Iran)	NR	240	72	NR	40	Q, CE	Moderate
Borhan Haghighi A ^6^	2013	Cross-sectional	Asia (Iran)	NR	40	62.5	37.2	7	Q, CE, EDT	Moderate
Hodacova L ^18^	2014	Cross-sectional	Europe (Czech)	2010-2011	575	28	46.4	84	Q	Moderate
Jaoude SB ^19^	2017	Cross-sectional	Asia (Lebanon)	2014	314	58.6	39.2	24	Q	Moderate
de Jesus Júnior LC ^9^	2018	Cross-sectional	America (Brazil)	2014	286	50	NR	38	Q	Moderate
Alhusain FA ^1^	2019	Cross-sectional	Asia (Saudi Arabia)	2017	223	60	NR	17	Q	High
Meisha DE ^22^	2019	Cross-sectional	Asia (Saudi Arabia)	NR	234	54.3	NR	21	Q	Moderate
Ohlendorf D ^26^	2020	Cross-sectional	Europe (Germany)	2018-2019	450	36.2	35	14	Q	Moderate
Maghsoudipour M ^21^	2021	Cross-sectional	Asia (Iran)	NR	106	37	NR	19	Q, CE, EDT	High

NR: not reported; Q: questionnaire; CE: clinical examination; EDT: electrodiagnostic test.

### Prevalence of CTS among dentists

**Figure 2.**
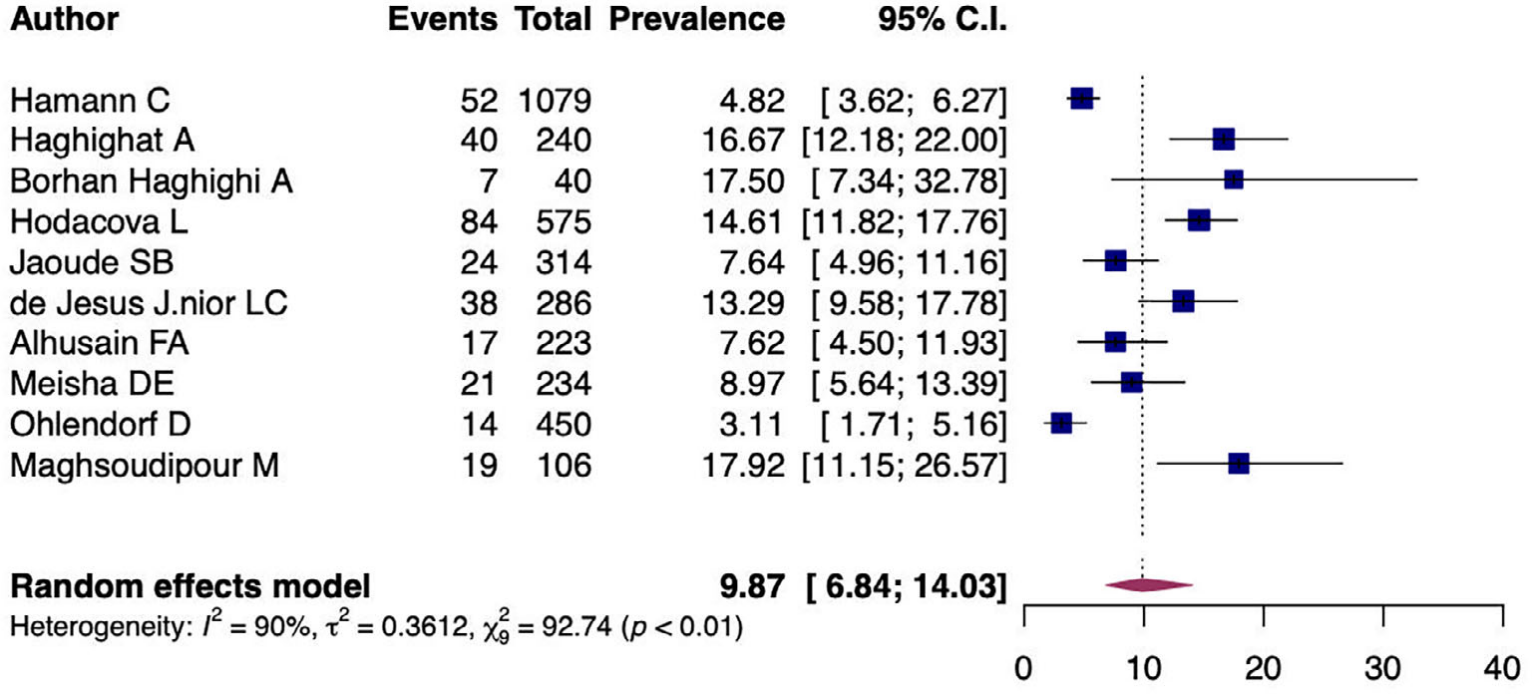
Forest plot evaluating the calculated prevalence of CTS among dentists using random-effects model.

**Figure 3.**
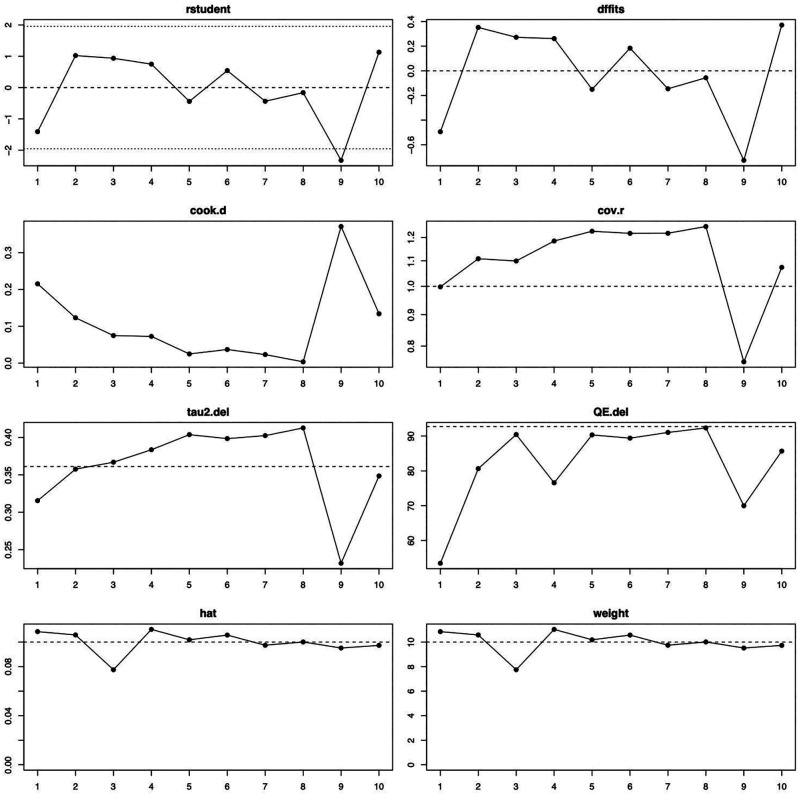
Visual representation of the influence diagnostics for each of the included studies. Abbreviations used—rstudent: studentized deleted residuals; dffits: DFFITS values; cook.d: Cook’s distances; cov.r: covariance ratio; tau2.del: estimated τ2 values; QE.del: estimated Cochran’s Q values.

**Figure 4.**
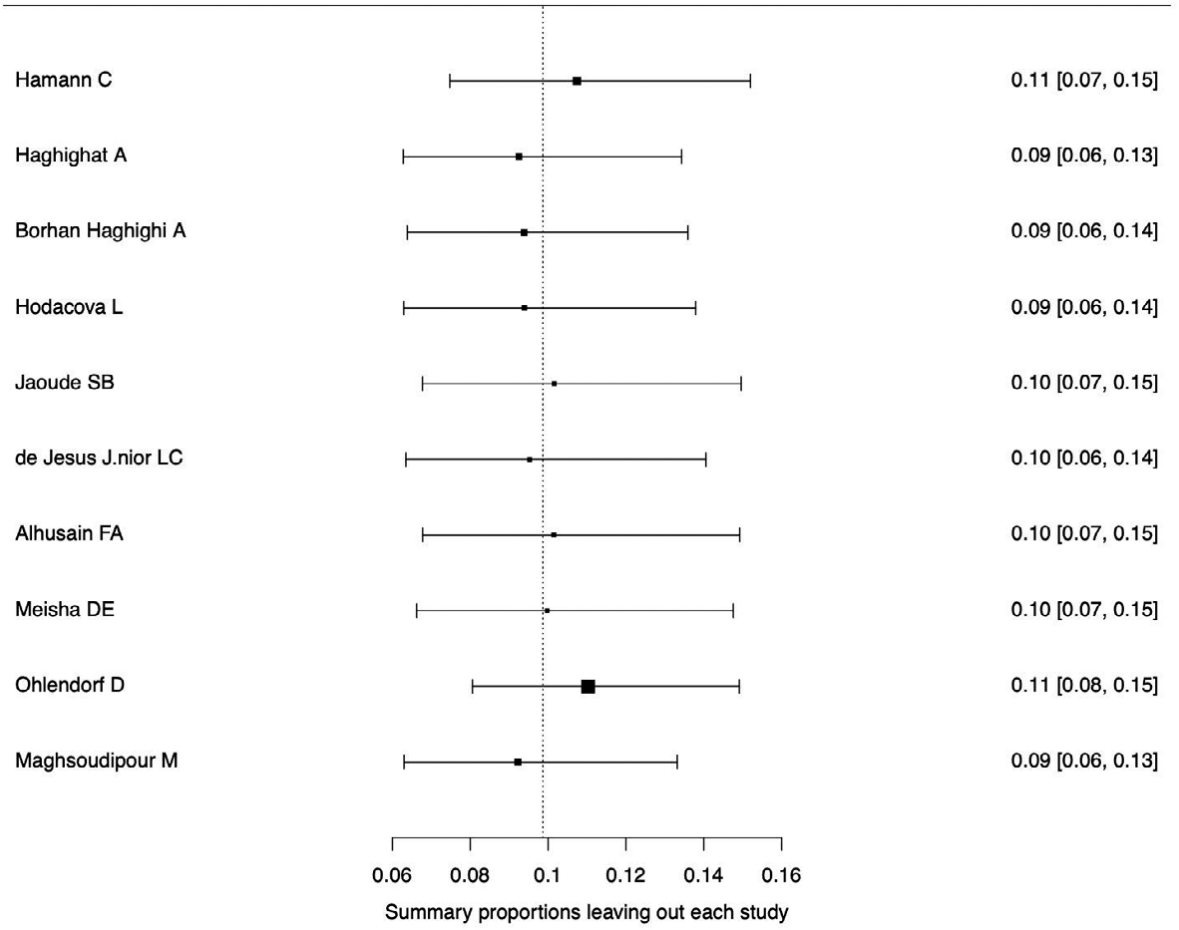
Forest plot displaying the re-calculated pooled effects, with one study omitted each time, using the leave-one-out method.

A random-effects model analysis yielded an initial overall CTS prevalence of 9.87% (95%CI 6.84%-14.03%) with significant heterogeneity between studies I ^2^=90.55% (95%CI 79.29%-97.31%, p<0.01) ( Figure 2). The influence diagnostics are presented in Figure 3. The forest plot illustrating the results of the leave-one-out analysis is presented in Figure 4. As per them, no study was identified as being influential. In other words, there was no study identified that was capable of turning the effect of the analysis into some direction.

### Moderator analysis

**Figure 5.**
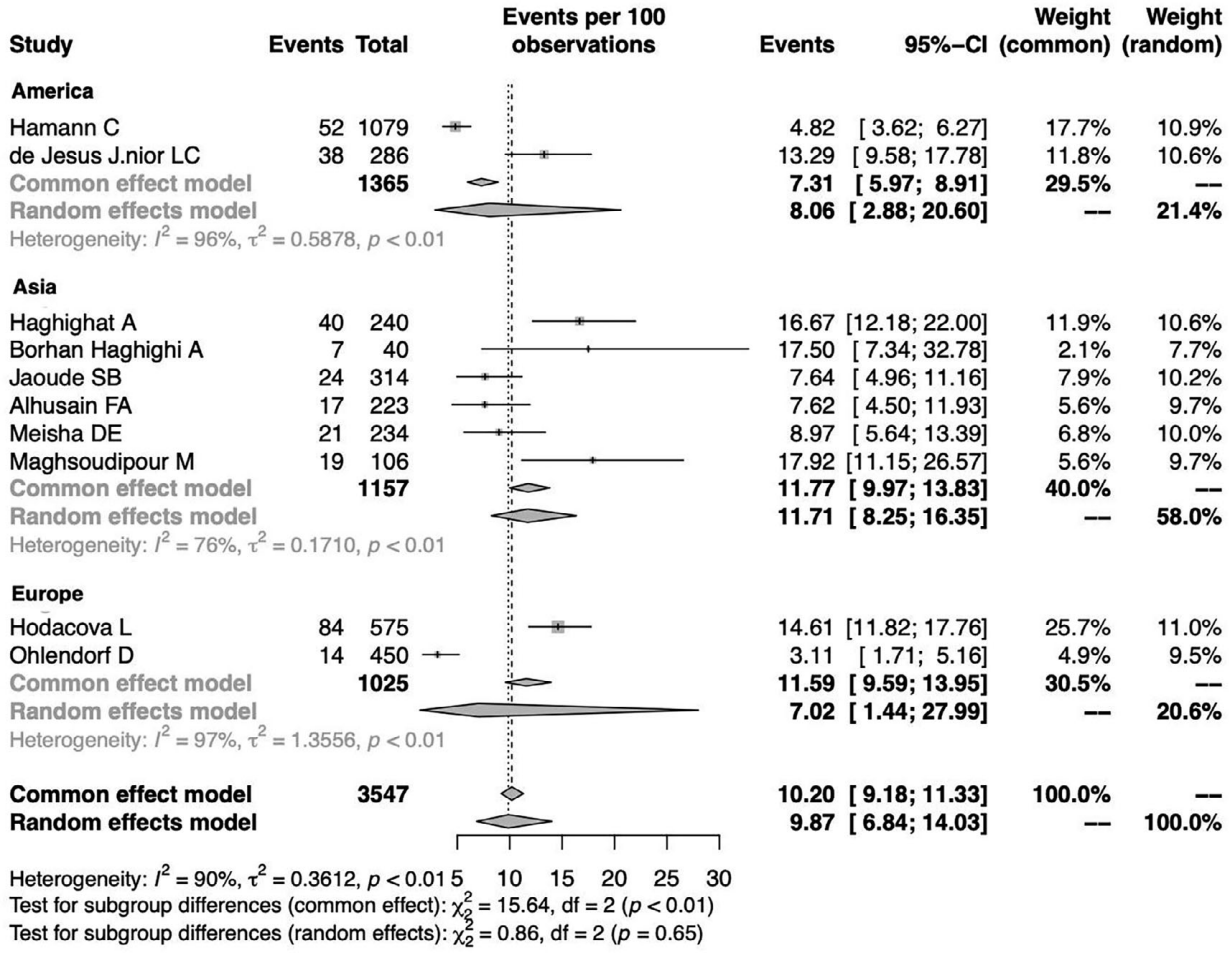
Forest plot illustrating the prevalence of CTS among dentists by continent of origin: America, Asia, Europe.

**Figure 6.**
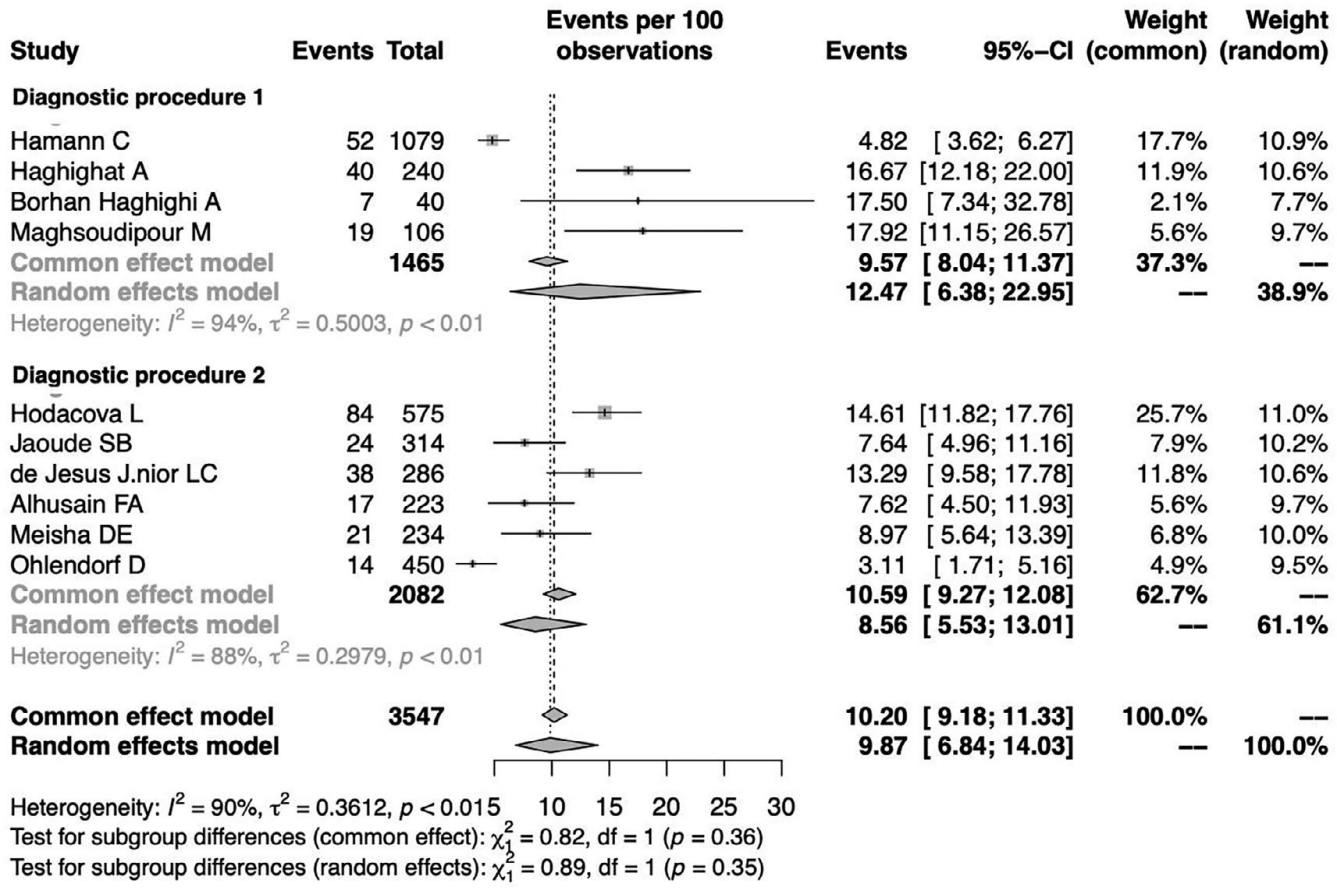
Forest plot illustrating the prevalence of CTS among dentists by diagnostic procedure (Diagnostic procedure 1: questionnaire, clinical examination, electrodiagnostic test; Diagnostic procedure 2: questionnaire).

To investigate the effect of potential risk factors in the heterogeneity, a moderator analysis was performed. Forest plots of the subgroup analysis are illustrated in Figure 5 and Figure 6. The prevalence was 7.02% (95%CI 1.44%-27.99%) among studies conducted in Europe, 8.06% (95%CI 2.88%-20.60%) among studies conducted in America and higher among those conducted in Asia (11.71%) (95%CI 8.25%-16.35%). The prevalence was 12.47% (95%CI 6.38%-22.95%) for the group identified as having CTS through medical history and at least clinical examination or electrodiagnostic testing and 8.56% (95%CI 5.53%-13.01%) among those who identified solely through questionnaire (previously diagnosed, self-reported). Heterogeneity remained high in the subgroup analysis by both continent of origin and type of diagnostic procedure. In the meta-regression analysis with continuous variables, the year of publication and the proportion of males, no statistically significant (positive or inverse) modification was found as presented in Table 2.

**Table 2.** Meta-regression analysis.

	K	QM	Regression coefficient	p-value
Year of publication	10	76.57	0.02 (95%CI -0.05-0.08)	0.63
Proportion of males	10	0.26	-0.01 (95%CI -0.03-0.02)	0.61

K=number of studies.

## Discussion

CTS is one of the most frequently diagnosed entrapment neuropathy, accounting for high disability among different occupations. ^24^ To date, only systematic reviews regarding musculoskeletal disorders (which is a general term referring to injuries in muscles, ligaments, tendons, nerves, blood vessels, bones and joints) among dental healthcare providers exist in the scientific literature. One indicative example of the above is the meta-analysis conducted by Chenna *et al*, in which the authors combined data from 88 studies and found out that seven out of ten dental healthcare workers (including dentist, dental students, dental hygienist and dental auxiliaries) experienced a musculoskeletal disorder. As per the location of the disorders, the most affected sites were the neck, the back, the lower back, the shoulder, the upper back and the wrist with a prevalence of 51%, 50%, 46%, 41%, 35% and 31%, respectively. ^7^

To the best of our knowledge, this is the first attempt to calculate the prevalence of CTS among dentists, through a systematic review. We do not have previously published data to compare our pooled estimate with. The prevalence of the existing observational studies varies considerably in the scientific literature. Our study provides evidence for 9.87% (95%CI 6.84%-14.03%) prevalence of CTS among dentists. Overall, the results are based on highly heterogeneous articles. Through the moderator analysis, we do not manage to identify sources of heterogeneity between the eligible studies. In the subgroup analysis, the prevalence was 12.47% (95%CI 6.38-22.95) for the group identified as having CTS through medical history and at least clinical examination or electrodiagnostic testing while, the prevalence was 8.56% (95%CI 5.53%-13.01%) among those who identified solely through questionnaire (previously diagnosed, self-reported). It should be noted that the latter pooled estimate may underestimate the dental surgeons with CTS due to the diagnostic method used. In matter of other oral health care professionals, Anton D., *et al*, found an 8.4% prevalence of CTS among 95 dental hygienists ^3^ while, Cherniack M., *et al*, calculated a 14.9% prevalence among 94 dental hygienists. ^8^ In a recent meta-analysis, Epstein S., *et al*, combining data from seven eligible studies, found a 9% (95%CI 5%-16%) prevalence of CTS among 2449 physicians (from different specialties including general surgeons, plastic surgeons, orthopedic surgeons and urologists) with significant heterogeneity between studies I ^2^=94.5%. ^11^ All the aforementioned results align with our estimation, providing more evidence that CTS can be considered as an occupational hazard among health care professionals.

It should be noted that there are many treatments available for this entrapment neuropathy. Patients developing mild or moderate symptoms should be treated conservatively through splinting, local corticosteroid injection or oral prednisone. Other treatments available, such as physical therapy, have not proven their effectiveness yet. Surgical decompression is the treatment of choice for patients developing severe symptoms. ^16^^,^^35^ Our study reports a considerable prevalence, consequently, the importance of awareness among dentists, regarding the etiology of this issue is more than necessary. More research should be conducted in order to explore the association between CTS among dentists and potential risk factors, such as gender, obesity, endocrine conditions (hypothyroidism, acromegaly and diabetes) and trauma.

### Study’s strengths and limitations

The main strength was the comprehensive methodology applied for literature search, study selection, specific inclusion/exclusion criteria, screening for eligibility, quality assessment and pooling analysis of prevalence data from ten studies. Nonetheless, the present study had several limitations. It should be noted that the unidentified heterogeneity remained on high levels, therefore, the results should be interpreted with caution. The highly heterogenous outcomes across the included studies were expected due to the nature of this type of studies. Owing to the limited availability of data (less than ten studies for each covariate) regarding variables such as mean age, obesity, diabetes, hypothyroidism, pregnancy, autoimmune diseases, these data were not included in this analysis. Lastly, only observational studies written in English language were included resulting in the occurrence of reporting bias.

## Conclusion

In conclusion, the prevalence of CTS among dentists is estimated at 9.87% (95%CI 6.84%-14.03%). Our results were based on highly heterogeneous studies. Sources of heterogeneity were not identified. Our findings point to several directions for future research. Therefore, further studies, both prospective and retrospective need to be conducted in order this issue to be fully investigated.

## Data Availability

Figshare: Main characteristics and data outcome of the included studies. https://doi.org/10.6084/m9.figshare.22087427.v1. ^37^ Figshare: PRISMA_2020_checklist.pdf. figshare. https://doi.org/10.6084/m9.figshare.22069034.v1. ^38^ Data are available under the terms of the Creative Commons Zero “No rights reserved” data waiver (CC0 1.0 Public domain dedication).
