# Supplementary material for: Prevalence of carpal tunnel syndrome among dentists: a systematic review and meta-analysis
Source: F1000Res. 2023 Jul 31;12:196. Originally published 2023 Feb 20. [Version 3] doi: 10.12688/f1000research.131173.3 (PMC10442586; doi:10.12688/f1000research.131173.3)
Supplement: Peer review history

# Peer review: Reviewer response for version 3

**DOI:** 10.5256/f1000research.153668.r192513

**Published:** 2023-08-21

**Authors:** Mohammad Alavinia

**Affiliations:** 1 KITE, Toronto Rehabilitation Institute – University Health Network, University Health Network, Toronto, ON, Canada

Thank you for providing the opportunity to review the revised version. The authors answered all comments and concerns.

Are the rationale for, and objectives of, the Systematic Review clearly stated?

No

Is the statistical analysis and its interpretation appropriate?

Yes

Are sufficient details of the methods and analysis provided to allow replication by others?

Yes

Are the conclusions drawn adequately supported by the results presented in the review?

Yes

Reviewer Expertise:

SR and Metaanalysis.

I confirm that I have read this submission and believe that I have an appropriate level of expertise to confirm that it is of an acceptable scientific standard.

# Peer review: Reviewer response for version 2

**DOI:** 10.5256/f1000research.145508.r185139

**Published:** 2023-07-24

**Authors:** Mohammad Alavinia

**Affiliations:** 1 KITE, Toronto Rehabilitation Institute – University Health Network, University Health Network, Toronto, ON, Canada

Thank you for providing me with the opportunity to review the Systematic Review and Meta-analysis paper titled "Prevalence of carpal tunnel syndrome among dentists: a systematic review and meta-analysis.  

 This systematic review and meta-analysis were conducted to estimate the prevalence of carpal tunnel syndrome (CTS) among dental surgeons and the effect of possible moderators on it. As I do not work in this field, I need to be made aware of the importance of CTS in this occupation. However, using the explanation about CTS in the introduction, the prevalence of this problem is high among dental surgeons. Although the authors provide the reason for the high prevalence of this disease among surgeons, the reason for conducting the SR and MA is unclear. There is no reasonable justification for conducting this study. I would like to see the heterogeneity in the studies in this field, which shows the necessity for conducting this systematic review. I cannot understand how the results will add to the present knowledge of CTS among dental surgeons and the benefits of the results in practice. The results could be applied in daily practice by adding these points to the introduction and discussion. On the other hand, the audience for this study could be more precise for me. Are the audiences physicians involved in treating these patients, or dental surgeons, who should be aware of this occupational problem and do the preventive interventions themselves? 

 In addition to these points, some methodologic aspects should be considered.

 First: in the PRISMA figure, in the first stage, in addition to the duplicates, the authors mentioned that those 24 were also excluded due to other reasons. In the footnote, other reasons include reviews, clinical trials and .... How is it possible to determine clinical trials, reviews and ... before title and abstract?

 In PRISMA, authors mentioned that they retrieved 51 articles in Pubme, while in the text, they searched Medline. PubMed is the public library of Medline, and although these two search engines are similar but are not the same. As far as I know, the most popular search engine for Medline is OVID. I believe that the search is not comprehensive. Some important search engines are missing, such as PsycINFO and CINAHL. 

 I think adding two subtitles and inclusion and exclusion criteria is better.

 The first three lines of the results are methods, and better to explain them in the method section.

 Cross-sectional studies are specific studies that measure the prevalence of a study. So, I think if the aim is to find the prevalence of CTS, the authors should include just C/S studies and explain this in the method section.

 Please explain the diagnostic graphs and their application in MA in statistical analysis.

 Some grammar and typo errors need special attention. For example, line one of the results should be " **the manuscripts**." Or et al should be written as **et al.**, or the first paragraph of discussion line five and sic, dentist, and hygienist should be pleural.

Are the rationale for, and objectives of, the Systematic Review clearly stated?

No

Is the statistical analysis and its interpretation appropriate?

Yes

Are sufficient details of the methods and analysis provided to allow replication by others?

Yes

Are the conclusions drawn adequately supported by the results presented in the review?

Yes

Reviewer Expertise:

SR and Metaanalysis.

I confirm that I have read this submission and believe that I have an appropriate level of expertise to confirm that it is of an acceptable scientific standard, however I have significant reservations, as outlined above.

# Response

**Published:** 2023-07-25

**Authors:** Ευάγγελος Κωσταρές

Dear Reviewer,

 On behalf of all authors, we would like to thank you for your kind words and valuable input regarding our manuscript.

 Please find a point-to-point response to each of your comments.

**Comment #1**

 This systematic review and meta-analysis were conducted to estimate the prevalence of carpal tunnel syndrome (CTS) among dental surgeons and the effect of possible moderators on it. As I do not work in this field, I need to be made aware of the importance of CTS in this occupation. However, using the explanation about CTS in the introduction, the prevalence of this problem is high among dental surgeons. Although the authors provide the reason for the high prevalence of this disease among surgeons, the reason for conducting the SR and MA is unclear. There is no reasonable justification for conducting this study. I would like to see the heterogeneity in the studies in this field, which shows the necessity for conducting this systematic review. I cannot understand how the results will add to the present knowledge of CTS among dental surgeons and the benefits of the results in practice. The results could be applied in daily practice by adding these points to the introduction and discussion. On the other hand, the audience for this study could be more precise for me. Are the audiences physicians involved in treating these patients, or dental surgeons, who should be aware of this occupational problem and do the preventive interventions themselves?

**Response #1**

 Thank you for your remark. We greatly appreciate your feedback and understand your concerns regarding the necessity of our systematic review and meta-analysis on the prevalence of carpal tunnel syndrome (CTS) among dental surgeons.

 Firstly, regarding the necessity of conducting a meta-analysis in this field, it is essential to consider that individual studies might provide varying estimates of CTS prevalence due to differences in study designs, populations, and methodologies. By conducting a systematic review and meta-analysis, we aimed to pool data from multiple studies to obtain a more precise and robust estimate of CTS prevalence among dental surgeons. The use of a meta-analysis is particularly relevant in cases where studies exhibit heterogeneity, as it helps to explore potential sources of variation and enhances the statistical power to detect significant associations.

 Secondly, we believe it is crucial to inform dental surgeons about the high prevalence of CTS in their profession due to its occupational significance. The repetitive and forceful hand movements involved in dental procedures can lead to an increased risk of developing CTS. By raising awareness of this issue, dental surgeons can better understand the importance of ergonomics and the implementation of preventive measures in their practice. Simple adjustments in posture, instrument handling, and regular breaks can significantly reduce the risk of developing CTS and other related musculoskeletal disorders. Furthermore, our study's results add value by providing an evidence-based assessment of CTS prevalence among dental surgeons, which can serve as a foundation for future research and guideline development. By informing dental surgeons and healthcare professionals involved in treating CTS patients, our findings encourage proactive measures to mitigate the impact of CTS on the dental workforce and improve the overall well-being and long-term health of dental professionals. Nevertheless, a comprehensive statement has been incorporated into the discussion section.

**Comment #2**

 First: in the PRISMA figure, in the first stage, in addition to the duplicates, the authors mentioned that those 24 were also excluded due to other reasons. In the footnote, other reasons include reviews, clinical trials and .... How is it possible to determine clinical trials, reviews and ... before title and abstract?

**Response #2**

 Thank you for your thoughtful review of our PRISMA figure. We appreciate your keen observation regarding the exclusion of 24 articles in the first stage. As you correctly pointed out, in addition to duplicates, these articles were excluded due to other reasons, including reviews, clinical trials, and more.

 Regarding your question on how it is possible to determine clinical trials, reviews, and other categories before reading the title and abstract, we agree that it would be challenging to do so manually. However, in PubMed, many articles indicate their classification, such as clinical trials, in the footnotes. This information can be valuable for initial filtering without having to read the entire title or abstract. Additionally, PubMed offers filters that allow users to specifically select clinical trials, reviews, and other types of articles, streamlining the search process and enabling quick exclusion of irrelevant articles from the beginning.

**Comment #3**

 In PRISMA, authors mentioned that they retrieved 51 articles in Pubme, while in the text, they searched Medline. PubMed is the public library of Medline, and although these two search engines are similar but are not the same. As far as I know, the most popular search engine for Medline is OVID. I believe that the search is not comprehensive. Some important search engines are missing, such as PsycINFO and CINAHL.

**Response #3**

 Thank you for your remark. The PRISMA (Preferred Reporting Items for Systematic Reviews and Meta-Analyses) guidelines are essential for conducting high-quality systematic reviews and meta-analysis. One of the fundamental principles emphasized by PRISMA is the need for a comprehensive search strategy to identify all relevant studies. Regarding your point about the differences between PubMed and Medline, we completely agree. While PubMed includes the Medline database, they are not exactly the same. Medline is a comprehensive bibliographic database of life sciences and biomedical literature, while PubMed is a free search engine that accesses the Medline database and also includes additional content ( https://pubmed.ncbi.nlm.nih.gov/about/). We chose to focus on PubMed and Scopus as these databases are widely recognized and widely used in the medical and scientific community. We understand your concern about the search potentially being incomplete by excluding other important databases such as PsycINFO and CINAHL. However, if the topic of a review directly pertains to the primary focus of specialized subject databases, like CINAHL for nursing and allied health or PsycINFO for behavioral sciences and mental health, they should indeed be added to ensure a comprehensive search (DOI: https://doi.org/10.1186/s13643-017-0644-y).

 By clearly stating the databases used and the search strategy employed, we aimed to uphold the highest standards of academic integrity. This practice helps to establish a strong foundation for our findings, enabling readers and fellow researchers to assess, validate, and build upon our work with confidence. Keeping a transparent approach ensures that our contributions to the scientific community are robust and reliable.

 In conclusion, our research was conducted using two prominent databases, namely PubMed and Scopus, supplemented by examining the reference lists of the available articles. The decision to utilize two databases was based on the understanding that such an approach enhances both coverage and recall, thus reducing the likelihood of overlooking relevant studies (DOI: https://doi.org/10.1016/j.jclinepi.2022.05.022).

**Comment** **#4**

 The first three lines of the results are methods, and better to explain them in the method section.

**Response #4**

 Thank you for your remark. The relevant amendments have been performed.

**Comment #5**

 Cross-sectional studies are specific studies that measure the prevalence of a study. So, I think if the aim is to find the prevalence of CTS, the authors should include just C/S studies and explain this in the method section.

**Response #5**

 Thank you for your remark. You rightly pointed out that cross-sectional studies are specific types of research that measure the prevalence of a particular condition or characteristic within a population at a specific point in time. We agree that these studies can be valuable in determining the prevalence of CTS among dentists.

 In our article, we aimed to investigate the prevalence rates of CTS among dentists specifically. To achieve this objective, we included observational studies written in the English language with no restriction on publication date. By employing this broad inclusion criteria, we aimed to gather comprehensive and up-to-date information on the prevalence of CTS in the dental profession.

 While cross-sectional studies are indeed useful for measuring prevalence, they are not the only study design that can provide valuable insights into this topic. By including various types of observational studies, such as cohort and case-control studies, we sought to capture a more comprehensive view of CTS prevalence, potential risk factors, and associations with dentistry-related factors.

 In the Method section of our article, we did clarify the inclusion criteria, explaining that we aimed to encompass all relevant observational studies that examined the prevalence rates of CTS among dentists. This broader approach allowed us to explore the topic from multiple angles and provide a more robust understanding of the prevalence and potential factors associated with CTS in the dental community.

 We acknowledge that cross-sectional studies alone can offer certain advantages, such as assessing prevalence in a specific time frame. However, combining different study designs can enhance the overall quality of evidence and allow for a more well-rounded discussion of the topic.

**Comment #6**

 Please explain the diagnostic graphs and their application in MA in statistical analysis.

**Response #6**

 Thank you for your remark. The statistical analysis section has been revised accordingly.

**Comment #7**

 Some grammar and typo errors need special attention. For example, line one of the results should be " **the manuscripts**." Or et al should be written as **et al.**, or the first paragraph of discussion line five and sic, dentist, and hygienist should be pleural

**Repsonse #7**

 Thank you for your remark. The relevant amendments have been performed.

 We hope that our response addresses your concerns, and we genuinely appreciate your engagement with our research. If you have any further suggestions or questions, please don't hesitate to let us know.

 Best regards,

 The corresponding author

# Peer review: Reviewer response for version 2

**DOI:** 10.5256/f1000research.145508.r166950

**Published:** 2023-03-29

**Authors:** Essam Ahmed Al‐Moraissi

**Affiliations:** 1 Department of Oral and Maxillofacial Surgery, Thamar University, Dhamar, Yemen

The authors have addressed my comments appropriately.

Are the rationale for, and objectives of, the Systematic Review clearly stated?

No

Is the statistical analysis and its interpretation appropriate?

Partly

Are sufficient details of the methods and analysis provided to allow replication by others?

No

Are the conclusions drawn adequately supported by the results presented in the review?

No

Reviewer Expertise:

Oral and Maxillofacial Surgery

I confirm that I have read this submission and believe that I have an appropriate level of expertise to confirm that it is of an acceptable scientific standard.

# Peer review: Reviewer response for version 1

**DOI:** 10.5256/f1000research.143986.r163987

**Published:** 2023-03-06

**Authors:** Essam Ahmed Al‐Moraissi

**Affiliations:** 1 Department of Oral and Maxillofacial Surgery, Thamar University, Dhamar, Yemen

This systematic review (SR) assessed the prevalence of CTS among dentists. The study is of interest. However, there are some major revisions that need to be made as follows:

 Abstract: The authors should clearly state the inclusion criteria, as well as the predictor and outcome variables in the abstract. The results of the risk of bias assessment should be included in the results section and not in the conclusion.

 Introduction: The authors should state the rationale for the study and summarize the results of previous studies on CTS.

 Method: This SR should be prepared in accordance with PRISMA guidelines and should follow the PRISMA checklist (which is mandatory). The inclusion criteria should be based on PICOS criteria. The authors should clearly define how CTS was diagnosed in the included studies. If the authors estimated proportions or used subgroup analyses, the synthesis of results (statistics) should be clearly explained.

Are the rationale for, and objectives of, the Systematic Review clearly stated?

No

Is the statistical analysis and its interpretation appropriate?

Partly

Are sufficient details of the methods and analysis provided to allow replication by others?

No

Are the conclusions drawn adequately supported by the results presented in the review?

No

Reviewer Expertise:

Oral and Maxillofacial Surgery

I confirm that I have read this submission and believe that I have an appropriate level of expertise to state that I do not consider it to be of an acceptable scientific standard, for reasons outlined above.

# Response

**Published:** 2023-03-13

**Authors:** Ευάγγελος Κωσταρές

Dear Reviewer,

 On behalf of all authors, we would like to thank you for your kind words and valuable input regarding our manuscript.

 Please find a point-to-point response to each of your comments.

**Comment #1**

 This systematic review (SR) assessed the prevalence of CTS among dentists. The study is of interest.

**Response #1**

 Thank you for your remark. Yet, please note that our attempt exceeds the spectrum of a systematic review by utilizing statistical methods (meta-analysis) to summarize the results of the studies identified through the systematic review of the available literature.

**Comment #2**

 Abstract: The authors should clearly state the inclusion criteria, as well as the predictor and outcome variables in the abstract. The results of the risk of bias assessment should be included in the results section and not in the conclusion.

**Response #2**

 Thank you for your comment. Taking into consideration your input, the abstract has been reformed accordingly. Yet, if additional modifications should be performed, please provide us with some guidance to amend the relevant section as soon as possible.

**Comment #3**

 Introduction: The authors should state the rationale for the study and summarize the results of previous studies on CTS.

**Response #3**

 Thank you for your comment. Taking into consideration your input, the rationale, the results of other studies as well as the aim of the current study have been altered and stated in the two last paragraphs of the introduction. Yet, please note that within this section, the results of other studies on CTS are only being reported as findings, since they are being analyzed in the results and discussion section later within the same document as well as given that they are not “systematic” or “meta-analytic” data.

**Comment #4**

 Method: This SR should be prepared in accordance with PRISMA guidelines and should follow the PRISMA checklist (which is mandatory).

**Response #4**

 Thank you for your comment. Please be aware that without any change of the original manuscript, the requested information can be found in two sections of the manuscript: *(a) the first line of the Methods *and *(b) The Data availability – Underlying data. *Additionally, the PRISMA flow chart is illustrated as Figure 1. Yet, in case there are additional requirements set by the PRISMA guidelines that oblige the authors on reporting additional information that are not being included, please, provide us with a response to conform our manuscript as soon as possible.

**Comment #5**

 The inclusion criteria should be based on PICOS criteria.

**Response #5**

 Thank you for your comment. To the best of our knowledge, the PICO(S) criteria, are being used in a way of formulating one or several research questions (Aslam, S., & Emmanuel, P. (2010). Formulating a researchable question: A critical step for facilitating good clinical research. *Indian journal of sexually transmitted diseases and AIDS*, *31*(1), 47–50. https://doi.org/10.4103/0253-7184.69003).

 Therefore, their main use is related to the basis on which the whole research will be made while the inclusion criteria, are more associated with the methodology that will be followed for data identification, selection and extraction given that different criteria may apply in various occasions (such as in times where language restrictions exist). In such scenario, manuscripts that would be included in the original pool can be excluded if these restrictions are valid and are being reported in the methodology to produce a repetitive result. Therefore, even if not mentioned, our inclusion criteria were based on PICO(S) criteria and their selection was not only justified through them but through the methodology that was used as reported in “Criteria for study selection and data extraction” subsection. Yet, if additional information should be included, please provide us with some guidance to amend the relevant subsection as soon as possible.

**Comment #6**

 The authors should clearly define how CTS was diagnosed in the included studies.

**Response #6**

 Thank you for your comment. Please be aware that without any change of the original manuscript, the requested information can be found  in Table 1 (Column 10 – Diagnosis). Nevertheless, a brief explanation has been included in “Search results and characteristics of the included studies” subsection (line 6-8).

**Comment #7**

 If the authors estimated proportions or used subgroup analyses, the synthesis of results (statistics) should be clearly explained.

**Response #7**

 Thank you for your comment. Please be aware that without any change of the original manuscript, the requested information can be found in the Statistical Analysis and Results sections. To the best of our understanding, by re-evaluating the provided in-text explanations, the methodology that was used (that may be referred to as the standard one utilized for the conduction of proportional meta-analyses) as well as the content of other meta-analyses available in the literature (in form of a brief comparison), we strongly believe that the information reported in this manuscript are adequate to enable a reader with a medium or less experience in meta-analyses to understand the approach that was followed. Yet, in case you believe that additional information should be included, please, provide us with a response to conform our manuscript as soon as possible.

 Hopefully, our changes as well as the responses provided above will be sufficient to accept our effort.

 Best regards,

 The corresponding author
